# Targeted Re-Sequencing Identified rs3106189 at the 5′ UTR of TAPBP and rs1052918 at the 3′ UTR of TCF3 to Be Associated with the Overall Survival of Colorectal Cancer Patients

**DOI:** 10.1371/journal.pone.0070307

**Published:** 2013-08-05

**Authors:** Jiaofang Shao, Xiaoyan Lou, Jun Wang, Jing Zhang, Chen Chen, Dasong Hua, Fan Mo, Xu Han, Shu Zheng, Biaoyang Lin

**Affiliations:** 1 Cancer Institute (Key Laboratory of Cancer Prevention and Intervention, China National Ministry of Education), Second Affiliated Hospital, College of Medicine, Zhejiang University, Hangzhou, Zhejiang Province, China; 2 Collaborative Innovation Center for Diagnosis and Treatment of Infectious Diseases and State Key Laboratory for Diagnosis and Treatment of Infectious Diseases, The First Affiliated Hospital of Medical College, Zhejiang University, Hangzhou, China; 3 Systems Biology Division and Propriumbio Research Center, Zhejiang-California International Nanosystems Institute (ZCNI), Zhejiang University, Hangzhou, Zhejiang Province, China; 4 Swedish Medical Center, Seattle, Washington, United States of America; 5 Department of Urology, University of Washington, Seattle, Washington, United States of America; Sapporo Medical University, Japan

## Abstract

Recent studies have demonstrated the power of deep re-sequencing of the whole genome or exome in understanding cancer genomes. However, targeted capture of selected genomic whole gene-body regions, rather than the whole exome, have several advantages: 1) the genes can be selected based on biology or a hypothesis; 2) mutations in promoter and intronic regions, which have important regulatory roles, can be investigated; and 3) less expensive than whole genome or whole exome sequencing. Therefore, we designed custom high-density oligonucleotide microarrays (NimbleGen Inc.) to capture approximately 1.7 Mb target regions comprising the genomic regions of 28 genes related to colorectal cancer including genes belonging to the WNT signaling pathway, as well as important transcription factors or colon-specific genes that are over expressed in colorectal cancer (CRC). The 1.7 Mb targeted regions were sequenced with a coverage ranged from 32× to 45× for the 28 genes. We identified a total of 2342 sequence variations in the CRC and corresponding adjacent normal tissues. Among them, 738 were novel sequence variations based on comparisons with the SNP database (dbSNP135). We validated 56 of 66 SNPs in a separate cohort of 30 CRC tissues using Sequenom MassARRAY iPLEX Platform, suggesting a validation rate of at least 85% (56/66). We found 15 missense mutations among the exonic variations, 21 synonymous SNPs that were predicted to change the exonic splicing motifs, 31 UTR SNPs that were predicted to occur at the transcription factor binding sites, 20 intronic SNPs located near the splicing sites, 43 SNPs in conserved transcription factor binding sites and 32 in CpG islands. Finally, we determined that rs3106189, localized to the 5′ UTR of antigen presenting tapasin binding protein (TAPBP), and rs1052918, localized to the 3′ UTR of transcription factor 3 (TCF3), were associated with overall survival of CRC patients.

## Introduction

With 639,000 deaths per year worldwide, colorectal cancer is the third most common form of cancer and the second leading cause of cancer-related deaths in the Western world (WHO, February 2009, http://www.who.int/mediacentre/factsheets/fs297/en/) and in China [1], [2]. To date, susceptibility to colorectal cancer has been characterized by the identification of rare inherited mutations in a small number of established genes such as mutations of the *APC* gene, a gene first identified as the familial adenomatous polyposis (FAP) locus gene [3] that contributes to colorectal tumorigenesis [1], [4]. SNPs (single nucleotide polymorphisms) are the most frequent type of variation in the human genome, occurring once every several hundred base pairs throughout the genome [5].

Recent studies have demonstrated the potential power of deep re-sequencing of candidate genes in human populations to detect rare variants and aid in the understanding of complex human traits [6]. Traditionally, cancer genome re-sequencing has been performed using exon amplification and conventional Sanger sequencing [7]–[9]. More recently, the whole genome or whole exome (by exome capturing) has been used due to technological advances and reduced cost in next generation sequencing [10]–[12]. For example, Bass *et al.* applied whole genome sequencing to sequence the tumors of 9 CRC patients and identified 11 in-frame gene fusion events including the fusion of VTI1A and TCF7L2, which was found in 3 of 97 colorectal cancers [13]. The Cancer Genome Atlas Network recently performed exome capture DNA sequencing of colorectal cancers and identified frequently mutated genes including APC, TP53, KRAS, PIK3CA, FBXW7, SMAD4, TCF7L2, NRAS, ARID1A, SOX9 and FAM123B (WTX) genes [14].

Moreover, instead of capturing the whole exome, targeted capture of selected genes of interest will reduce cost and potentially move NGS into clinical practice. For example, Pritchard *et al.* developed Coloseq, in which selected regions of 1.1 Mb of DNA including 209 kb in *MLH1*, *MSH2*, *MSH6*, *PMS2*, *EPCAM*, *APC*, and *MUTYH* were targeted, captured and subjected to NGS [15]. The authors were able to identify 28/28 (100%) pathogenic mutations in MLH1, MSH2, MSH6, PMS2, EPCAM, APC, and MUTYH [15].

We were interested in the targeted capture of genomic regions including the promoters and intronic regions of genes related to a pathway or a network of genes with certain characteristics to understand cancer biology. There are several advantages to this approach: 1) the genes can be selected based on biology or a hypothesis; 2) mutations in promoter and intronic regions, which have recently been suggested to have important regulatory roles, can be investigated; and 3) the technique is less expensive than whole genome or whole exome sequencing. Therefore, we designed custom high-density oligonucleotide microarrays (NimbleGen Inc.) to capture a total of approximately 1.7 Mb target regions comprising the genomic regions of 28 genes related to colorectal cancer including the exonic, intronic, 10 kb upstream and 5 kb downstream sequences followed by analysis using the Illumina Genome Analyzer. The selected genes include those belonging to the WNT signaling pathway, as well as important transcription factors or colon-specific genes that are over expressed in CRC.

## Results

### Targeted Re-sequencing of Genomic Regions Including Promoters of the Key WNT Pathway and Other CRC-related Genes

As the WNT signaling pathway is a critical pathway implicated in CRC [16], we selected two WNT pathway genes (http://www.genome.jp/kegg/pathway/hsa/hsa04310.html) to begin our investigation. In addition, we selected 22 important transcription factors (transcription regulator activity GO:0030528) and four colon-specific or enriched genes [17] that are over expressed in cancer based on data generated in the laboratory as well as data available in the public domain (e.g. GSE8671, GSE15960, GSE24551, GSE41258 from the GEO database). The final list of the selected 28 genes is shown in Table 1 with annotations.

**Table 1:** The sequence coverage of 28 selected genes and number of SNPs in CRC and CRN identified by NGS.

				NG coverage (%)^a^		total coverage (%)^b^		folds coverage (x)^c^		no. SNPs^d^			SNP rate (‰)^e^		
Gene	Entrez Gene ID	Chromosome Location^f^	Descriptions	CRC	CRN	CRC	CRN	CRC	CRN	CRC	CRN	Comb.	CRC	CRN	Comb.
APC	324	chr5∶112063584–112186935	Adenomatosis polyposis coli	119.2	119.3	99.87	99.99	46	46	135	151	166	1.094	1.224	1.346
AXIN1	8312	chr16∶327440–407464	Axin 1	132.8	136.4	95.45	98.07	19	40	49	72	90	0.612	0.9	1.125
DAXX	1616	chr6∶33276335–33295791	Death-associated protein 6	134.6	138.9	95.68	98.76	22	39	8	19	20	0.411	0.977	1.028
ETS2	2114	chr21∶40167231–40201879	V-ets erythroblastosis virus E26 oncogene homolog 2 (avian)	112.0	112.4	98.9	99.29	45	54	78	73	88	2.251	2.107	2.54
ETV4	2118	chr17∶41595212–41628762	Ets variant gene 4 (E1A enhancer binding protein, E1AF)	121.8	123.0	99.05	100	29	48	32	45	47	0.954	1.341	1.401
FLI1	2313	chr11∶128553989–128687310	Friend leukemia virus integration 1	106.3	106.8	99.53	100	45	57	222	237	265	1.665	1.778	1.988
FUBP1	8880	chr1∶78390603–78449777	Far upstream element (FUSE) binding protein 1	119.4	119.5	99.99	100	46	47	61	63	87	1.031	1.065	1.47
GTF2A2	2958	chr15∶59921065–59954719	General transcription factor IIA, 2, 12 kDa	134.5	135.5	98.71	99.44	39	45	73	89	99	2.169	2.644	2.942
HIPK2	28996	chr7∶139247375–139426847	Homeodomain interacting protein kinase 2	116.5	116.9	83.47	83.8	35	46	134	138	176	0.764	0.787	1.003
HMGA1	3159	chr6∶34194650–34219008	High mobility group AT-hook 1	107.7	115.2	88.37	94.52	22	39	13	21	24	0.534	0.862	0.985
KAT5	10524	chr11∶65469489–65492074	K(lysine) acetyltransferase 5	110.9	111.2	95.28	99.29	25	40	8	13	16	0.354	0.576	0.708
LRTOMT	220074	chr11∶71798368–71819433	leucine rich transmembrane and 0-methyltransferase domain containing	145.2	145.9	99.82	100	35	57	24	30	32	1.139	1.424	1.519
MDM2	4193	chr12∶69191980–69239214	Mdm2, transformed 3T3 cell double minute 2, p53 binding protein (mouse)	103.7	104.9	99.16	99.64	34	39	26	29	40	0.55	0.614	0.847
MSX2	4488	chr5∶174141575–174162901	Msh homeobox 2	117.6	119.4	98.47	99.67	31	48	29	31	38	1.36	1.454	1.782
NR3C1	2908	chr5∶142647496–142820077	Nuclear receptor subfamily 3, group C, member 1 (glucocorticoid receptor)	138.0	139.8	98.1	99.61	45	46	184	278	333	1.066	1.611	1.93
PPARA	5465	chr22∶46536499–46644653	Peroxisome proliferator-activated receptor alpha	123.8	123.8	98.19	99.49	29	44	44	62	78	0.407	0.573	0.721
PRKCDBP	112464	chr11∶6330176–6346740	Protein kinase C, delta binding protein	145.7	149.1	99.84	100	32	42	19	30	35	1.147	1.811	2.113
RNF25	64320	chr2∶219518588–219541781	Ring finger protein 25	108.9	112.2	99.96	100	35	52	11	15	19	0.474	0.647	0.819
SMARCA4	6597	chr19∶11061606–11177953	SWI/SNF related, matrix associated, actin dependent regulator of chromatin, subfamily a, member 4	131.7	132.0	97.11	99.35	20	42	55	78	90	0.473	0.67	0.774
SOX4	6659	chr6∶21583972–21603847	SRY (sex determining region Y)-box 4	130.3	136.6	94.54	97.33	35	43	17	15	24	0.855	0.755	1.207
STAT5A	6776	chr17∶40429565–40468958	Signal transducer and activator of transcription 5A	122.8	124.8	94.91	99.52	20	41	25	35	37	0.635	0.888	0.939
TCF3	6929	chr19∶1599293–1655277	Transcription factor 3 (E2A immunoglobulin enhancer binding factors E12/E47)	121.4	124.9	95.71	98.43	12	35	108	138	153	1.929	2.465	2.733
TFDP1	7027	chr13∶114229056–114300499	Transcription factor Dp-1	120.6	121.6	98.27	99.1	32	52	27	45	50	0.378	0.63	0.7
TNKS2	80351	chr10∶93548069–93630032	Tankyrase, TRF1-interacting ankyrin-related ADP-ribose polymerase 2	142.9	148.9	97.93	99.48	42	45	34	48	57	0.415	0.586	0.695
TRIM28	10155	chr19∶59045836–59067082	Tripartite motif-containing 28	129.5	149.6	85.28	98.48	17	39	65	81	105	3.059	3.812	4.942
VAV1	7409	chr19∶6762722–6862371	Vav 1 guanine nucleotide exchange factor	171.7	175.0	96.43	98.32	21	37	73	87	116	0.733	0.873	1.164
YBX1	4904	chr1∶43138072–43173073	Y box binding protein 1	116.3	117.4	98.04	98.97	45	52	25	25	31	0.714	0.714	0.886
ZNF3	7551	chr7∶99651851–99684363	Zinc finger protein 3	166.8	169.3	98.14	99.61	28	44	21	20	26	0.646	0.615	0.8

a. NG coverage (%): the percentage of regions covered by final reads out of the whole NimbleGen captured regions for each gene, including 10-kb upstream and 5-kb downstream.

b. total coverage (%): the percentage of regions covered by final reads out of the whole designed regions for each gene.

c. folds coverage (x): the average read depth.

d. no. SNPs: the total SNPs identified for the gene.

e. SNP rate (‰): the average count of SNP in a 1k-bp window.

f. The chromosome names and locations of the genomic regions that were captured for each gene.

To reduce expenses, we first sequenced a pool of 30 CRC tissues (the CRC pool) and a pool of 30 adjacent normal tissues (the CRN pool) and then validated the SNPs identified using PCR or Sequenom’s technologies. We created a custom oligo array using NimbleGen technology to capture the target sequences. The total length of the target genomic regions designed was 1.7 Mbp. The captured DNAs were subjected to sequencing using the Illumina Genome Analyzer. After removing PCR duplicates from the raw sequences, the average coverage ranged from 32x to 45x, and the coverage by sequence length for the targeted regions of each gene ranged from 83.5 to 100%. The coverage for the different regions of the target genes differed, which might be due to the property of NimbleGen sequence capture technology, sequence complexity or other uncharacterized factors. The raw sequencing data was deposited in the NCBI sequence read archive (SRA) under accession number SRX277359.

We tabulated the coverages of all 28 genes by comparing to regions covered by the designed probes or to the total targeted regions including promoters and 3′ distal regions (Table 1) to calculate the capture efficiency of the NimbleGen approach. Measured by the targeted regions, the median coverages was 98.1 and 99.5% for the CRC and the CRN tissues respectively, and ranging from 83.5 to 100% (Table 1). In the NinbleGen probe design, the probes were not designed as overlapping oligos to cover the complete regions, but rather as probes that spaced among the target regions with specific characteristics optimized to DNA capture. The coverage calculated by the regions covered by the designed probes all exceed 100% (Table 1), suggesting that the capture probes captured adjacent sequences in addition to their complementary sequences, which resulted in that the sequenced regions actually extended beyond the regions that were covered by the probes.

**Figure 1:**
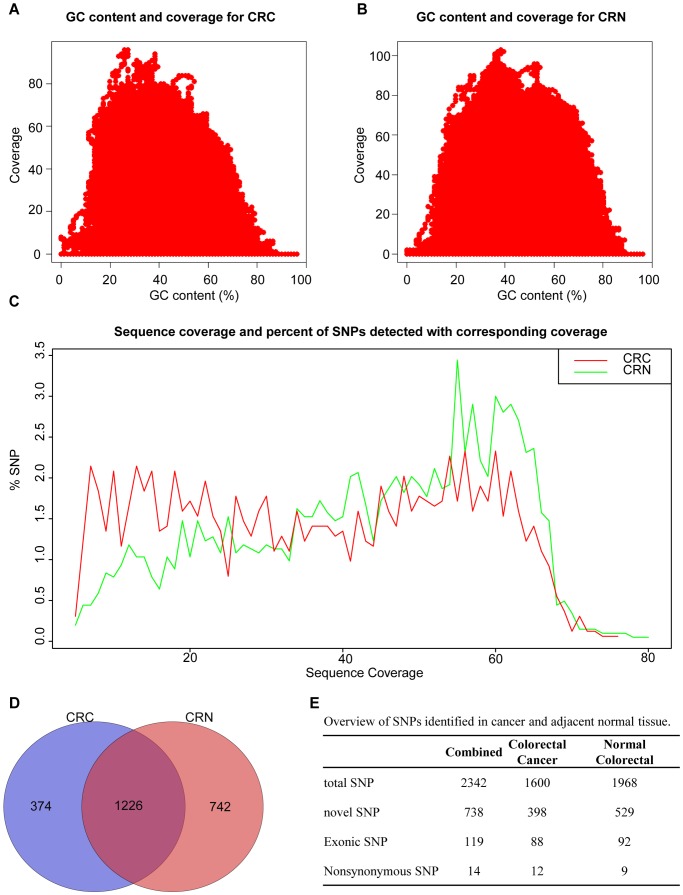
GC content, coverage and SNP count. (A) The GC content and coverage in CRC (colorectal cancer) tissue. (B) The GC content and coverage in CRN (colorectal normal tissue) tissue. (C) The relationship between sequence coverage and SNP detection. Red line shows the sequence coverage and percentage of SNPs detected at that coverage in CRC pool, and green line in CRN pool (D) Venn diagram of SNPs for CRC and CRN samples. (E) An overview of SNPs identified in cancer and adjacent normal tissue.

The GC content was computed for each position of the reference sequences centered in an 81-bp window in order to investigate whether the coverages were affected by the GC content of the captured regions. The coverage for each position was counted after removing duplicate sequences. Sufficient coverage of >40X was achieved for regions with a GC content between approximately 15–75% (Figure 1A, 1B). We next studied whether the difference in the coverage affected the detection frequency of sequence variations. We computed the Spearman correlation for the SNP count and the corresponding coverage using R (www.r-project.org). Here, the coverage was counted after removing sequence duplicates. The correlation coefficients were −0.51 and −0.38 for CRC and CRN samples, respectively, suggesting little correlation between SNP detection and read coverage. We further computed whether the SNP percentage accounted for the total SNPs with different coverages (Figure 1C). We found that the detection frequency remained flat when the sequence coverage increased from 40X to 60X for the CRC tissues. However, we found that the detection frequency in the normal tissue pools increased when the sequence coverage reached approximately 55X to 65X (Figure 1C). These differences might suggest a higher heterogeneity among the normal tissue pool than the CRC tissue pool, which may be explained by a similar tumor biology or mutation profiles among the CRC tissues. The detecting frequency dropped when the sequence coverage was greater than 65X, likely due to false high coverage generated for the repeated sequences for these regions.

After data analysis, we identified a total of 2342 sequence variations in the CRC and corresponding adjacent normal tissues. Among them, 738 were novel sequence variations based on comparisons with the current SNP database (dbSNP135; Table S1). 1226 variations were common to the CRC and normal colon tissues, while 374 and 742 variations were unique to each tissue type respectively (Figure 1D).

For the two pooled samples, the frequency of mutation rate ranged from 0.354 to 4.942 per kilobase for different genes. Most variations occurred in the intronic regions, with only 5% of the variations occurring in the exonic regions.

We randomly selected eight SNPs for validation covering variations found in intronic and in exonic regions. For validation, we used allele-specific PCR (AS-PCR) for genotyping single nucleotide polymorphisms [18], [19]. Each SNP was analyzed individually with a gene specific primer pair in a separate cohort of 22 CRC samples and 24 CRC adjacent normal tissues from the corresponding patients and four healthy donors (Table S5). We found that the data for four of the SNPs were consistent between the sequencing data and the PCR validation. For example, the SNPs for the MSX2 and KAT5 were detected 100% by the sequencing-based approach and by PCR validation. For rs80186078 in the TFDP1 gene, we only detected the SNP in the CRC tissues by sequencing and validating it in both CRC and CRN tissues but not in healthy donors by the AS-PCR validation. However, we also observed an inconsistency between the sequencing of the pooled samples and the PCR validation of individual samples. For example, rs11186694 and rs17107140 were detected in both CRC and CRN samples by sequencing but could not be detected by AS-PCR in individual samples. This result suggests a false positive identification of SNPs or a failure of the AS-PCR. We did not attempt to design additional PCR primers for AS-PCR, as we determined that AS-PCR was cumbersome and lacked sensitivity [20]. Furthermore, some of the SNPs (e.g., chr11∶65481267_TG) were detected in one pooled sample but were found in both CRC and normal tissues when analyzed by PCR validation of individual samples. This result suggests a false negative identification of SNPs in one of the pooled samples. However, it might not be surprising because if the allele frequency of the SNPs is low in one of the pooled samples, it might be missed by sequencing of pooled samples.

Due to the low efficiency and sensitivity of SNP validation by PCR, we decided to use the Sequenom MassARRAY iPLEX Platform for the validation studies. We chose 66 SNPs for validation in a separate cohort of 30 CRC tissues because the DNA used for sequencing was depleted. In the end, we were able to confirm the existence of 56 SNPS in the 30 CRC tissues (Table S6), suggesting a validation rate of at least 85% (56/66), considering that some of the detection failures might be due to differences in the sample population.

### Functional Consequence of the Identified Sequence Variations

We found 15 SNPs that would change protein sequences among the exonic variations in the CRC and normal colon tissues, including 14 missense mutations and 1 nonsense mutation (Figure 1E and Table 2). These missense mutations may affect the function of the mutated protein products. The novel SNP chr13∶114288328_CT identified only in CRC tissues would result in a stop codon, which would cause early termination of the translation of TFDP1 (NP_009042, Q200*) and loss of the Transc_factor_DP_C domain in the truncated TFDP1 protein. The effect of this truncated TFDP1 on CRC carcinogenesis remains to be investigated.

**Table 2:** PolyPhen, SIFT and PROVEAN prediction results for non-synonymous variations identified.

SNP position	SNP ID	Protein	Acc. no.	Variation	Sample^a^	PolyPhen	SIFT (cutoff = 0.05)	PROVEAN (cutoff = −2.5)	Validated by Sequenom^b^	Previously identified as CRC mutation?
chr1∶78392446_GA	rs1166698	NEXN	NP_653174	G245R	CRC; CRN	Probably damaging	Damaging	Neutral	+	No
chr11∶6340706_AG	rs1051992	PRKCDBP	NP_659477	L158P	CRC; CRN	Benign	Tolerated	Neutral	+	No
chr5∶174156371_GA	Novel	MSX2	NP_002440	A197T	–; CRN	Probably damaging	Damaging	Deleterious	−	No
chr5∶142779488_AG	Novel	NR3C1	NP_000167	F306S	CRC; –	Probably damaging	Tolerated	Neutral	−	No
chr5∶112176756_TA	rs459552	APC	NP_000029	V1822D	CRC; CRN	Benign	Tolerated	Neutral	−	Yes. Confers a protective effect with an odds ratio of 0.76 (CI = 0.60–0.97) among colon patients.
chr6∶34214322_CG	rs1150781	C6orf1	NP_848603	G150A	CRC; CRN	Benign	Tolerated	Neutral	−	No
chr11∶65481267_TG	Novel	KAT5	NP_874369	V213G	CRC; –	Benign	Tolerated	Neutral	−	No
chr5∶174156168_TC	rs4242182	MSX2	NP_002440	M129T	CRC; CRN	Benign	Tolerated	Neutral	−	No
chr19∶1615796_GA	rs2074888	TCF3	NP_003191	A492V	–; CRN	Benign	Tolerated	Neutral	−	No
chr13∶114288328_CT	Novel	TFDP1	NP_009042	Q200*	CRC; –	N/A	N/A	N/A	−	No
chr6∶33283766_TC	rs3130100	ZBTB22	NP_005444	T310A	CRC; CRN	Benign	Tolerated	Neutral	−	No

a Tissue samples with SNP detected by NGS. CRC is the colorectal cancer tissue pool, and CRN is the colorectal cancer adjacent normal tissue pool.

b “+”indicates “validated” and “−” indicated “not tested” by Sequenom.

Four of the mutations failed to be validated by Sequenom’s MassARRAY iPLEX (Table S6) and were therefore excluded from further analysis. Four of the remaining 11 missense sequence variations identified in the CRC and normal colon tissues were novel mutations. The online tools PolyPhen, SIFT and PROVEAN were used to predict the functional consequences (Table 2). All three programs predicted that the novel mutations for MSX2 (A197T) would affect the functional domains of the protein and might have functional consequences. The NEXN (G245R) variation was predicted to have functional consequences by the SIFT and PolyPhen programs (Table 2). PolyPhen predicted another mutation in the NR3C1 gene to be likely damaging (Table 2). We also assessed whether these 11 mutations have been previously reported for CRC. Ten of them have not been previously reported to be associated with CRC and therefore were identified for the first time (Table 2). One of them, rs459552 in the APC gene has been reported to confer a protective effect for CRC with an odds ratio of 0.76 (CI = 0.60–0.97) among CRC patients [21].

There were 29 synonymous SNPs detected in the coding region in the CRC and CRN samples and 73 SNPs in the 5′ or 3′ UTR regions. FastSNP was used to predict the regulatory roles of these SNPs including exonic splicing enhancer (ESE), exonic splicing silencer (ESS), motif changes for synonymous SNPs (Table 3), and TF binding sites changes for UTR SNPs (Table 4). The ESE finder can identify ESEs recognized by individual SR proteins that are highly conserved splicing factors, and RESCUE-ESE can search sequences with ESE activity. In contrast, FAS-ESS can identify ESS. The prediction results from the three computational tools were combined to confirm whether the single nucleotide variation would change the splicing motif. The transcription factor binding sites associated with the target SNPs were identified by TFSEARCH using FastSNP. A total of 21 synonymous SNPs were predicted to change the exonic splicing motifs, and 31 UTR SNPs were predicted to occur at the transcription factor binding sites and therefore might affect gene transcription. The novel SNP chr2∶219524460_CA (5′UTR of BCSIL) was also found in conserved transcriptional binding sites (Table S2).

**Table 3:** List of synonymous SNPs with ESE/ESS motifs changed.

Gene	SNP ID	SNP position	Sample^a^	Validated by Sequenom^b^
DAXX	rs1059231	chr6∶33288271_AG	CRC;CRN	+
HIPK2	rs7456421	chr7∶139415775_CG	CRC;CRN	+
APC	rs2229992	chr5∶112162854_TC	CRC;CRN	−
APC	rs351771	chr5∶112164561_GA	CRC;CRN	−
APC	rs41115	chr5∶112175770_GA	CRC;CRN	−
APC	rs42427	chr5∶112176325_GA	CRC;CRN	−
APC	rs866006	chr5∶112176559_TG	CRC;CRN	−
AXIN1	rs1805105	chr16∶396264_AG	CRC;CRN	−
ETS2	rs457705	chr21∶40191431_TG	CRC;CRN	−
ETS2	rs461155	chr21∶40191638_AG	CRC;CRN	−
FUBP1	Novel	chr1∶78422291_TG	CRC;–	−
NR3C1	Novel	chr5∶142779439_AG	CRC;CRN	−
PDIA2	rs432925	chr16∶334580_GC	–;CRN	−
PPARA	rs150197646	chr22∶46611153_TC	CRC;–	−
PRKCDBP	rs12570	chr11∶6340525_AT	CRC;CRN	−
STAT5A	rs1135669	chr17∶40459737_CT	CRC;CRN	−
TFDP1	Novel	chr13∶114294549_CT	CRC;CRN	−
TFDP1	rs4150756	chr13∶114277541_CT	CRC;–	−
TNKS2	rs3758499	chr10∶93608142_GA	CRC;CRN	−
TRIM28	rs2305120	chr19∶59059729_GA	–;CRN	−
ZSCAN21	rs11558476	chr7∶99654689_GA	CRC;CRN	−

a Tissue samples with SNP detected by NGS. CRC is the colorectal cancer tissue, and CRN is the colorectal cancer adjacent normal tissue.

b “+”indicates “validated” and “−” indicated “not tested” by Sequenom.

**Table 4:** List of UTR SNPs with transcription factor binding sites changed using TFSEARCH.

Region	Gene	SNP ID	SNP position	Sample^a^	Validated by Sequenom^b^
3′UTR	ETS2	rs1051425	chr21∶40195485_TC	CRC;CRN	+
5′UTR	TAPBP	rs138551513	chr6∶33282002_CT	–;CRN	+
3′UTR	APC	rs41116	chr5∶112180921_TC	CRC;CRN	−
3′UTR	APC	rs448475	chr5∶112181379_CG	CRC;CRN	−
3′UTR	APC	rs397768	chr5∶112181576_GA	CRC;CRN	−
5′UTR	BCS1L	Novel	chr2∶219524460_CA	CRC;–	−
3′UTR	C19orf26	rs36074840	chr19∶1230677_GA	CRC;CRN	−
3′UTR	ETS2	rs711	chr21∶40195059_AG	CRC;CRN	−
3′UTR	ETS2	rs530	chr21∶40195277_TA	CRC;CRN	−
3′UTR	FLI1	rs682695	chr11∶128682460_CA	CRC;CRN	−
3′UTR	GTF2A2	rs8027421	chr15∶59930865_AT	–;CRN	−
3′UTR	GTF2A2	rs8027679	chr15∶59930772_GC	CRC;CRN	−
3′UTR	HIPK2	rs1638195	chr7∶139252780_TC	CRC;CRN	−
3′UTR	HMGA1	rs2780219	chr6∶34212743_AG	–;CRN	−
3′UTR	HMGA1	rs1150782	chr6∶34213868_AG	–;CRN	−
3′UTR	MDM2	Novel	chr12∶69237323_CT	–;CRN	−
3′UTR	MDM2	Novel	chr12∶69235967_GA	CRC;–	−
3′UTR	MDM2	rs1132585	chr12∶69237388_AG	CRC;CRN	−
3′UTR	MSX2	rs14459	chr5∶174157711_AG	CRC;CRN	+
3′UTR	MSX2	rs2890849	chr5∶174157762_GC	CRC;CRN	−
3′UTR	NEXN	rs3767028	chr1∶78408536_CG	–;CRN	−
3′UTR	RNASEH2C	rs535111	chr11∶65485337_AG	–;CRN	−
3′UTR	RNASEH2C	rs521678	chr11∶65485727_TG	CRC;CRN	−
3′UTR	STAT3	rs3744483	chr17∶40466438_TC	CRC;CRN	−
5′UTR	TAPBP	rs146763267	chr6∶33281842_GT	–;CRN	−
3′UTR	TCF3	rs41275834	chr19∶1609616_AT	CRC;CRN	−
3′UTR	TFDP1	Novel	chr13∶114294912_AT	–;CRN	−
3′UTR	TFDP1	Novel	chr13∶114295295_GA	–;CRN	−
3′UTR	TFDP1	Novel	chr13∶114294701_GT	CRC;–	−
3′UTR	TFDP1	Novel	chr13∶114295186_GA	CRC;–	−
5′UTR	ZNF142	rs4674324	chr2∶219523433_TG	CRC;CRN	−

a Tissue samples with SNP detected by NGS. CRC is the colorectal cancer tissue, and CRN is the colorectal cancer adjacent normal tissue.

b “+” indicates “validated” and “−” indicated “not tested” by Sequenom.

To understand the functional consequences of the intronic SNPs, the online tool SNPnexus was used to annotate the SNPs. The distances to the splicing sites were computed by SNPnexus. There were 20 intronic SNPs located near the splicing sites with a distance less than 30 bp, and only one was novel. The mutations at these regions may affect splicing and transcription. C6orf1, ETV4, KAT5 and VAV1 each had two variations located near splicing sites, and TNKS2 had 3 variations located near splicing sites (Table 5). The SNP rs2271959 (chr17∶41622740_GT, ETV4) was 5 bp away from the splicing site and was detected only in CRN tissues with high confidence. There were 43 intronic, upstream or intergenic SNPs in conserved transcription factor binding sites (Table S2) and 32 in CpG islands (Table S3).

**Table 5:** Intronic SNPs near splice sites (<30 nt).

SNP position	Gene	Acc. no.	Splice-dist. (bp)	SNP ID	Sample^a^	Validated by Sequenom^b^
chr17∶41622740_GT	ETV4	NM_001986	5	rs2271959	–;CRN	+
chr17∶41598940_TC	DHX8	NM_004941	7	rs2271957	CRC;CRN	+
chr19∶1624007_AG	TCF3	NM_003200	8	rs55677929	CRC;CRN	+
chr10∶93600480_GA	TNKS2	NM_025235	17	rs17107140	CRC;CRN	+
chr17∶41623212_GA	ETV4	NM_001986	17	rs79158595	–;CRN	+
chr10∶93572984_CT	TNKS2	NM_025235	20	rs11186694	CRC;CRN	+
chr21∶40193488_GA	ETS2	NM_005239	22	rs117862776	–;CRN	+
chr19∶59059798_CT	TRIM28	NM_005762	23	Novel	–;CRN	+
chr11∶65481166_TC	KAT5	NM_182710	28	rs1151500	CRC;CRN	+
chr11∶71804513_GA	LRTOMT	NM_145309	29	rs2250866	CRC;CRN	+
chr5∶112170870_TC	APC	NM_000038	8	rs62626346	–;CRN	−
chr19∶6822219_TC	VAV1	NM_005428	13	rs347033	–;CRN	−
chr5∶142680344_CA	NR3C1	NM_000176	16	rs6188	CRC;–	−
chr6∶34215228_GA	C6orf1	NM_178508	16	rs1150780	–;CRN	−
chr10∶93617306_AG	TNKS2	NM_025235	19	rs1340420	CRC;CRN	−
chr16∶359953_AG	AXIN1	NM_003502	20	rs2301522	–;CRN	−
chr2∶219527005_CT	BCS1L	NM_004328	22	rs2303561	–;CRN	−
chr6∶34215221_GA	C6orf1	NM_178508	23	rs928482	–;CRN	−
chr19∶6833989_GT	VAV1	NM_005428	25	rs308199	CRC;CRN	−
chr11∶65480791_AG	KAT5	NM_006388	28	rs551115	CRC;CRN	−

a Tissue samples with SNP detected by NGS. CRC is the colorectal cancer tissue, and CRN is the colorectal cancer adjacent normal tissue.

b “+” indicates “validated” and “−” indicated “not tested” by Sequenom.

The public ChIP-seq datasets, especially the ENCODE project, provide vast TF binding or DNAase hypersensitivity sites in various cell lines. Here, we used RegulomeDB to annotate the SNPs with regulatory regions. Each SNP was given a score that represented different regulatory regions by RegulomeDB (Table S1, Table 6). The aforementioned, likely damaging, missense SNP rs1166698 (NEXN, validate by Sequenom) received a score of 1b, which was the highest in this study, indicating that the SNP was involved in many important regulatory regions. Another 1b SNP was rs1860661, located in the intron of TCF3 and not tested by Sequenom. Among the 2342 SNPs, 1062 were situated in TF binding regions defined by ChIP-seq technology.

**Table 6:** Overview of RegulomeDB annotation.

Score	SNP Counts	Supporting data sets
1b	2	eQTL+TF binding+any motif+DNase Footprint+DNase peak
1d	4	eQTL+TF binding+any motif+DNase peak
1f	26	eQTL+TF binding/DNase peak
2a	10	TF binding+matched TF motif+matched DNase Footprint+DNase peak
2b	52	TF binding+any motif+DNase Footprint+DNase peak
2c	2	TF binding+matched TF motif+DNase peak
3a	47	TF binding+any motif+DNase peak
4	243	TF binding+DNase peak
5	676	TF binding or DNase peak
6	510	Other
7	770	No data

### Analysis of Associations between SNPs and Overall Survival Time

We chose nine SNPs (Table 7) that were validated by the Sequenom MassARRAY iPLEX technology and with allele heterozygosities of greater than 0.4 for analysis of the association between SNPs and CRC patient survival. We collected samples from a set of 117 patients with detailed clinical information for this analysis using the Sequenom MassARRAY iPLEX technology. The distribution of the 117 patients’ demographic and clinicopathologic characteristics are summarized in Table 8, and the genotype data are summarized in Table S7.

**Table 7:** The association of single SNP with CRC patient survival.

					death/total			dominant			additive		recessive	
Gene	ref	allele	SNP ID	Region (a.a. substitution)	WW	WV	VV	HR (95%CI)		p	HR (95%CI)	p	HR (95%CI)	p
TAPBP	C	C/T	rs3106189	5′UTR	20/65	12/42	1/7	0.2489404(0.0871363–0.7112003)	**0.009**		0.3289281(0.1279939–0.8453038)	**0.021**	0.7882852(0.0967128–6.425145)	0.824
TCF3	T	C/T	rs1052918	3′UTR	12/37	12/47	5/22	0.2802584(0.0926905–0.8473875)	**0.024**		0.5921091(0.2649582–1.323202)	0.201	1.076675(0.3393056–3.416473)	0.9
ETV4	A	A/G	rs79868029	intronic	14/46	13/47	6/13	1.746839(0.6486287–4.704459)	0.27		1.453808(0.7773533–2.718915)	0.241	1.720894(0.5184039–5.71268)	0.375
ETV4	G	A/G	rs79158595	intronic	15/46	11/45	6/14	1.238529(0.4701443–3.26273)	0.665		1.249898(0.6641922–2.352096)	0.489	1.676421(0.4922574–5.70918)	0.409
DHX8	T	C/T	rs71361531	intronic	14/45	13/54	7/14	1.722156(0.6399157–4.634706)	0.282		1.516384(0.8136795–2.825954)	0.19	2.006176(0.6380821–6.307564)	0.234
NEXN	G	A/G	rs1166698	coding(G|R)	14/37	15/54	3/18	0.7786868(0.2625501–2.309476)	0.652		0.8774619(0.3942656–1.952844)	0.749	1.002838(0.2277706–4.415333)	0.997
HIPK2	C	G/C	rs7456421	coding(V|V)	1/11	11/51	23/53	1.491145(0.2662636–8.350797)	0.649		1.507939(0.7196225–3.159823)	0.276	1.836416(0.6948774–4.853267)	0.22
STAT5A	T	C/T	rs1053023	3′ downstream	14/40	16/62	2/8	0.4510602(0.1703864–1.194082)	0.109		0.5324628(0.2144653–1.32197)	0.174	0.8564487(0.102282–7.171397)	0.886
ETS2	T	C/T	rs1051425	3′UTR	0/3	14/42	20/67	omit			2.06858(0.7675036–5.575248)	0.151	omit	?

Note: The significant P values (≤0.05) are in bold.

WW, homozygous wild-type genotype; WV heterozygous genotype; VV, homozygous variant genotype.

Abbreviations: CI, confidence interval; HR, hazard ratio; omit, no results due to missing information on the death status.

**Table 8:** The clinicopathological characteristics of 117 Chinese CRC patients.

Variables		Number of patients	% (n = 117)
Age	> = 60	71	60.68%
	<60	46	39.32%
Gender	Male	69	58.97%
	Female	48	41.03%
Tumor position	Colon	66	56.41%
	Rectum	51	43.59%
Tumor differentiation	Poor	4	3.42%
	Moderate	37	31.62%
	Well	53	45.30%
	Not available	23	19.66%
Infiltration depth	Outside of serosa	31	26.50%
	Serosa	47	40.17%
	Propria	21	17.95%
	Mucosa	2	1.71%
	Not available	16	13.68%
Positive lymph node	Null	60	51.28%
	>0	37	31.62%
	Not available	20	17.09%
Tumor stage	I	18	15.38%
	II	45	38.46%
	III	41	35.04%
	IV	13	11.11%

**Figure 2:**
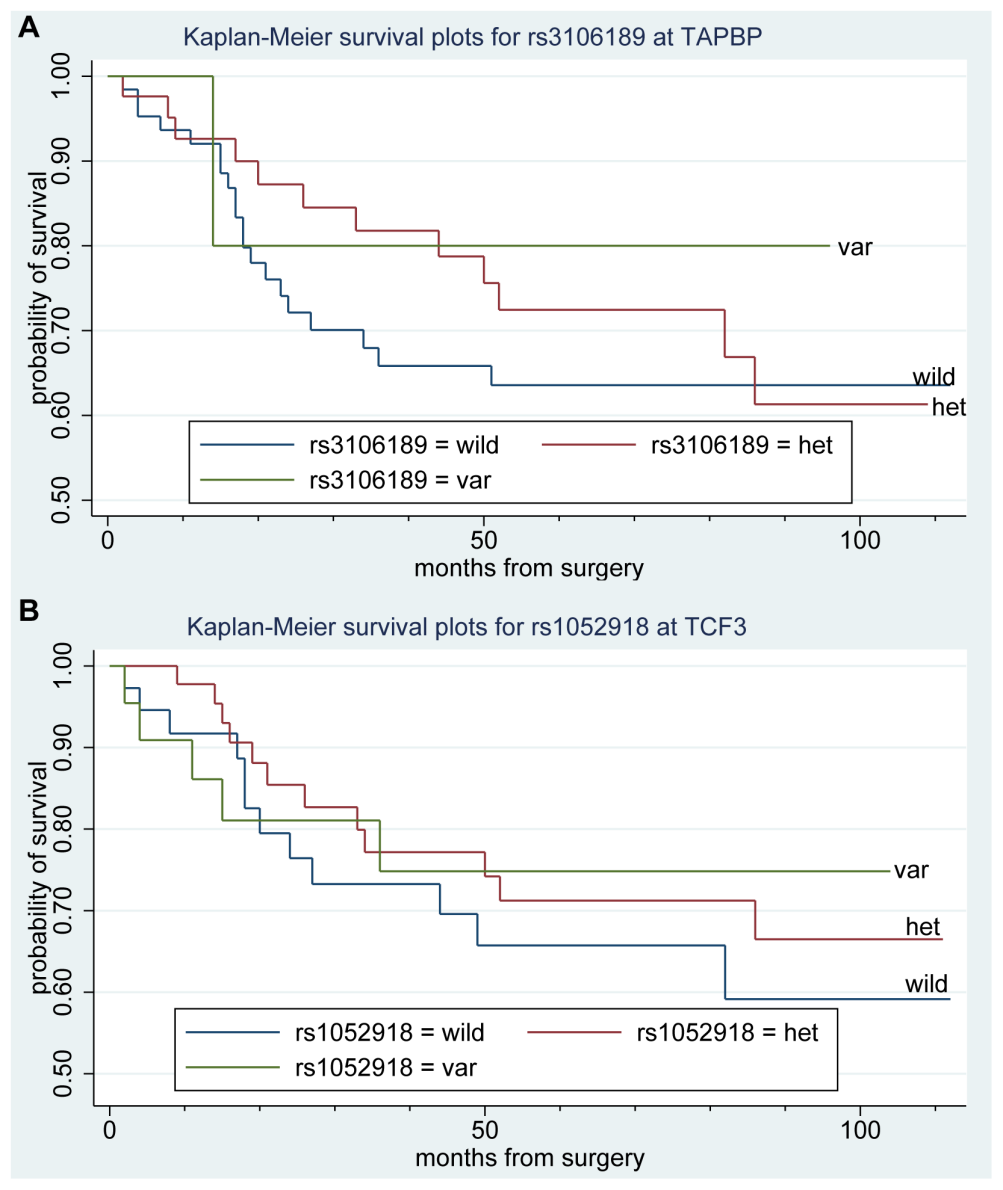
Colorectal cancer overall survival in relationship to SNPs. (A) Kaplan-Meier plot for rs3106189 localized to the 5′ UTR of TAPBP. (B) Kaplan-Meier plot for rs1052918 localized to the 3′ UTR of the TCF3. Y-axis, CRC survival probability; X-axis, months from surgery. Blue lines are homozygous wildtype (wild), green are homozygous variant (var), red are heterozygous variant (het).

We first analyzed the Hardy-Weinberg equilibrium of each SNP and found that only SNP rs1053023 deviated from the Hardy-Weinberg equilibrium (Table 9, p<0.05); the P values for other SNPs ranged from 0.3265 to 1. The effect of the nine SNPs on overall survival time was assessed in 117 CRC patients using the Kaplan-Meier method and plotted using the Stata 12 (www.stata.com) statistical analysis program. We found that two SNPs (rs3106189 and rs1052918) were associated with overall survival of CRC patients (Figure 2) using the dominant model with hazard ratios of 0.25 (P = 0.009) and 0.28 (P = 0.024), respectively. The SNP rs3106189 was also significantly associated with CRC patient survival with the additive model (hazard ratio = 0.33, P = 0.021; Table 7). The SNP rs3106189 localized to the 5′ UTR of TAPBP, and the SNP rs1052918 localized to the 3′ UTR of the TCF3. For the SNP rs3106189, the numbers of patients with heterozygous and homozygous variants were 42 and 7 respectively. For the SNP rs1052918, the numbers of patients with heterozygous and homozygous variants were 47 and 22 respectively. Patients bearing one of the two variants seem to have higher probabilities to survive longer.

**Table 9:** The Hardy-Weinberg equilibrium of the SNPs.

SNP ID	Gene Name	Pearson chi2	P-value	LR chi2	P-value	Exact Significance
rs1053023	STAT3	5.895	0.0152	6.185	0.0129	**0.0215**
rs1052918	TCF3	0.958	0.3276	0.958	0.3278	0.3265
rs1051425	ETS2	1.446	0.2291	1.588	0.2076	0.3963
rs79158595	ETV4	0.319	0.5724	0.317	0.5736	0.6663
rs79868029	ETV4	0.034	0.8527	0.034	0.8529	0.8319
rs71361531	DHX8	0.127	0.7215	0.128	0.7209	0.8395
rs3106189	TAPBP	0.004	0.9505	0.004	0.9506	1
rs1166698	NEXN	0.052	0.8193	0.052	0.8193	1
rs7456421	HIPK2	0.063	0.8013	0.064	0.8008	1

## Discussion

In this manuscript, we describe our analysis pipeline that consists of (1) initially sequencing pooled DNA samples followed by validation and further analysis in larger cohorts of samples for cost reduction and (2) a hypothesis-driven targeted capturing and analysis of SNPs and their associations with the cancer phenotypes. Pooling genomic DNAs for sequencing has the advantage of reducing sample preparation and sequencing costs. For example, capturing 30 individual samples would require using 30 capture arrays to perform hybridization and sample recoveries, which are tedious and may potentially introduce sample-to-sample variations during the sample preparation stage. Sequencing 30 individual samples would also be substantially more costly than sequencing one pool. Although it is possible to use barcoding and multiplexing reactions and sequencing to achieve similar sequence coverage at a similar cost to pooling samples, the sample preparation complexity would be substantially higher. In a recent GWAS analysis of type 1 diabetes (T1D) published in Science, Nejentsev *et al.* re-sequenced exons and splice sites of 10 candidate genes in DNA pools from 480 patients and 480 controls to identify causative type 1 diabetes (T1D) variants and then tested their disease association in over 30,000 participants [22]. The authors were able to identify four rare variants that independently lowered T1D risk [odds ratios, 0.51 to 0.74; P = 1.3×10(−3) to 2.1×10(−16)] in interferon induced with helicase C domain 1 (IFIH1) [22].

Another distinct feature of our analysis pipeline is that we sequenced the genomic regions that included exonic and intronic regions, i.e., the 10-kb promoter and the 5-kb downstream genomic regions of the selected genes. This method was in contrast with most studies that only analyzed the exonic sequences (exome capture) [23], [24]. It is important to include the promoter regions in the analysis, as SNPs in the promoter regions have been associated with tumorigenesis. For example, Bond *et al.* showed that a single nucleotide polymorphism in the MDM2 promoter could attenuate the p53 tumor suppressor pathway and accelerate tumor formation in humans [25]. Passarelli *et al.* showed that SNPs in the estrogen receptor beta promoter are associated with survival of postmenopausal women with CRC [26]. Polymorphisms in the UTR regions of genes have also been found to be related to cancer. For example, Zhang *et al.* found that a polymorphism in the 3′UTR region of insulin-like growth factor I (IGF1) gene predicts survival of non-small cell lung cancer in a Chinese population [27]. Hao *et al.* found that a SNP (rs3213245, −77T>C) in the XRCC1 gene 5′ UTR contributes to diminished promoter activity and increased risk of non-small-cell lung cancer [28]. We have identified and validated using Sequenom’s platform several SNPs that localized to the 5′ or 3′ UTR of the genes (Table S6). For example, rs3106189 of TAPBP and rs8041394 of GTF2A2 localized to 5′ UTRs, and rs1051425 of ETS2 and rs1052918 of TCF3 localized to 3′UTRs (Table S6). The functional significance of these SNPs remains to be determined.

We have chosen genes related to the WNT pathway, as the Cancer Genome Atlas Network found mutations in 16 different genes in the WNT pathways including APC, CTNNB1, FAM123B and TCF7L2 [14]. We extended the analysis of the WNT pathway genes to regions beyond the exome analyzed the Cancer Genome Atlas Network, and our approach has the potential to identify those mutations that modulate gene expression or splicing in additional to the identification of those structurally damaging mutations in the exons.

We identified a total of 2342 sequence variations in CRC and corresponding adjacent normal tissues. Among them, 738 were novel sequence variations based on comparison with the current SNP database (dbSNP135; Table S1). We chose 66 SNPs for validation in a separate cohort of 30 CRC tissues. We were able to confirm the existence of 56 SNPS in the 30 CRC tissues (Table S6), suggesting a validation rate of at least 85% (56/66), considering that some of detection failures might be due to differences in the sample population. This validation rate is in line with the published validation rate of 85.4% for NGS using the Illumina platform [29]. In addition, it has been reported that various validation platforms including the Sanger sequencing, Pyrosequencing, Sequenom MassArray or SNAPshot SNP Detection lack the sensitivity to confirm sequence variants identified by deep sequencing in tumors, which may be contaminated with DNAs from normal tissues or which may contain multiple clones [30].

We identified 14 missense exonic mutations in the CRC and normal colon tissues (Table 2). The SNP (G245R) at the NEXN gene (Nexilin; F actin binding protein) was predicted to have functional consequences. The roles of the NEXN gene in cancer have not yet been investigated. Two novel SNPs in the nuclear receptor subfamily 3, group C, member 1 (NR3C1) and lysine acetyltransferase 5 (KAT5) genes were found only in CRC tissues but not in normal colon tissues. KAT5 (also named TIP60 or HIV-1-Tat interactive protein) is a histone acetyl transferase (HAT), and it plays important roles in regulating chromatin remodeling and in DNA repair and apoptosis [31]. In colorectal cancers, KAT5 down regulation is associated with more advanced stages of colorectal cancer [32]. NR3C1 (alias, glucocorticoid receptor) was found to be epigenetically deregulated in colorectal tumorigenesis [33]. Furthermore, hypermethylated NR3C1 is a CRC gene with microsatellite instability [34]. These novel SNPs in the KAT5 and NR3C1 genes warrant confirmation, and additional functional studies are needed to assess the functional consequences of the mutations and their relationship to cancer, such as whether the SNPs would mimic the epigenetic regulations of these genes.

We also identified SNPs that might affect exon splicing because they localize to the ESE (exonic splicing enhancer) and ESS (exonic splicing silencer), which are critical in exon splicing. For example, we identified SNPs in the far upstream element (FUSE) binding protein 1 (FUBP1), peroxisome proliferator-activated receptor alpha (PPARA) and transcription factor Dp-1 (TFDP1) that might affect exon splicing for these genes, and these SNPs were found only in the CRC tissues (Table 3). Zhang *et al.* showed that a SNP (−195 C>T; dbSNP ID: rs1056932) that alters a potential binding site for an exonic splicing enhancer could affect the risk of non-Hodgkin lymphoma [35]. The functional consequences of the SNPs that localize to the ESE or ES sequences in FUBP1, PPARA and TFDP1 genes warrant further investigation.

We determined that rs3106189, localized at the 5′ UTR of TAP binding protein (tapasin; TAPBP), and rs1052918, localized at the 3′ UTR of the TCF3, were associated with overall survival of CRC patients (Table 7 and Figure 2) with hazard ratios reaching 0.28 (P = 0.024) and 0.33 (P = 0.021) respectively. These data suggest that these two variants confer protective effects for CRC patients. Interestingly, another variant that we identified, the rs459552 in the APC gene, was previously reported to confer a protective effect for CRC with an odds ratio of 0.76 (CI = 0.60–0.97) among CRC patients [21]. However, we did not analyze this SNP by the Sequenom technology and therefore could not assess whether the finding is also true in our data set.

TAPBP encodes a transmembrane glycoprotein that mediates the interaction between newly assembled major histocompatibility complex (MHC) class I molecules and the transporter associated with antigen processing (TAP) [36]. Downregulation of TAPBP expression has been observed for multiple cancers, including CRC, as an immune escape mechanism of human tumors [37]. Loss of TAPBP expression has been observed in 80% of high-grade intraepithelial neoplasia (HIN) compared with autologous colorectal mucosa, in 63% of primary adenocarcinomas in stage III and 79% of the matched lymph node metastases [38]. The ex vivo introduction of TAPBP expression in a murine lung carcinoma model increased surface MHC class I and restored susceptibility of tumor cells to antigen-specific cytotoxic T lymphocytes (CTL) killing [39]. The SNP rs3106189 is located within an H3K27Ac histone mark, which is often found near active regulatory elements, and within H3K9Ac and H3K4me3 marks (UCSC genome browser; Figure S1). Furthermore, rs3106189 is localized among binding sites for several transcription factors including interferon regulatory transcription factor 1 (IRF-1), IRF-2 and IRF-7. The exact functional consequence of the variant at the rs3106189 locus requires further study.

Transcription factor 3 (TCF3; E2A immunoglobulin enhancer binding factors E12/E47) is a member of the TCF/LEF transcription factor family that is central in regulating epidermal and embryonic stem cell identity and is involved in the WNT signaling pathway [40]. In breast cancer, TCF3 is involved in the regulation of breast cancer cell differentiation state and tumorigenicity [40]. Furthermore, overexpression of TCF3 is partially responsible for the butyrate-resistant phenotype of CRC because TCF3 suppresses the hyper-induction of Wnt activity by butyrate [41]. The functional consequence of the variant at rs1052918, which is localized at the 3′ UTR of the TCF3, remains to be determined.

## Materials and Methods

### Ethics Statement

Informed written consent was obtained from each patient, and the ethics committee of the Second Affiliated Hospital, College of Medicine and Zhejiang University approved all aspects of the study.

### Clinical Samples and Genomic DNA Isolation

Tissue samples were obtained from four control subjects (healthy individuals) and 30 colorectal cancer patients for the first cohort and 117 CRC patients for the validation study. All subjects were Chinese, and the samples were collected at the Second Affiliated Hospital, Zhejiang University. The DNA samples were isolated using the DNeasy Blood & Tissue Kit (QIAGEN Inc., Valencia CA) according to the manufacturer’s protocol.

### NimbleGen Sequence Capture

Genomic DNAs from 30 colon cancer patients were mixed in equal ratios and the DNA pools were captured on a custom NimbleGen 385K array following the manufacturer’s protocol (Roche/NimbleGen, Madison, WI) with modifications at the W. M. Keck Facility at Yale University. A custom tiling 385K sequence capture array targeting genomic sequence for all the 28 genes (including 10-kb upstream and 5-kb downstream regions) was designed and manufactured by Roche NimbleGen. Briefly, the genomic DNA sample was fragmented and hybridized to the custom NimbleGen Sequence Capture array. Unbound fragments were removed, and the target-enriched DNA was eluted and amplified.

The captured DNA was sheared through sonication, and adapters were ligated to the resulting fragments. The adapter-ligated templates were fractionated by agarose gel electrophoresis, and the fragments of the desired size were excised. The extracted DNA was amplifed using ligation-mediated PCR, purifed, and hybridized to the capture array at 42.0°C using the manufacturer’s buffer. The array was washed twice at 47.5°C and three times at room temperature using the manufacturer’s buffers. Bound genomic DNA was eluted using 125 mM NaOH for 10 min at room temperature, purifed, and amplifed through ligation-mediated PCR. The resulting fragments were purifed and subjected to DNA sequencing on the Illumina platform. Captured and non-captured amplifed samples were subjected to quantitative PCR to measure the relative fold enrichment of the targeted sequence.

### Sequence Analyses by the Next Generation Sequencing Technology

Amplified DNA samples were sonicated for 10 minutes (130 w, Cole-Parmer CPX 130, Illinois USA) to generate DNA fragments with an average size of 500 base pairs (bp). The fragments were further purified and concentrated with Qiaquick PCR purification spin columns (QIAGEN Inc., Valencia CA). Genomic fragments were end-repaired with a mixture of T4 DNA polymerase, Klenow DNA polymerase and T4 PNK (Promega, Madison, WI USA), and a 3′ overhang A was added using Klenow exo-enzyme (Promega, Madison, WI USA). The resultant fragments were ligated with the Illumina classical adapters by DNA T4 ligase (Promega, Madison, WI USA) according to Illumina’s protocol. Adapter-linked DNA fragments were separated by agarose gel electrophoresis, and the band between 150 and 200 bp was excised from the gel. The DNA fragments were extracted from the agarose slices using the Qiaquick Gel Extraction Kit (Qiagen Inc.). Extracted DNA was enriched by an 18-cycle amplification using Illumina’s universal adapters. The DNA fragments were purified and quantified and then sequenced for 40 cycles using Illumina’s protocol.

### Short Read Mapping and SNP Calling

Short-sequence reads were extracted from the image files with the Illumina GAPipeline 1.4.0 and mapped to the genome sequence using BWA (version 0.5.3) with default parameters. BWA is an efficient program for aligning relatively short nucleotide sequences against a long reference sequence allowing mismatches, thus detecting SNPs [42].

SNPs were identified by SAMtools (version 0.1.6), which migrated and improved various downstream data processing implemented in Maq/Maqview such as indexing, pileup, viewer and consensus caller. SAMtools generated the consensus sequence with the statistical model implemented in MAQ [43], [44]. Potential PCR duplicates were first removed by the command “samtools rmdup”. Raw variations were called by the command “samtools pileup” with default parameters and then filtered by the command “samtools.pl varFilter” with default options except for the following: minimum read depth (-d 5) to filter out low covered regions, maximum read depth (-D 255) to filter out randomly placed repetitive hits. Those SNPs with an SNP quality greater than 45 were considered high quality and were used for subsequent analysis.

### Detecting Regions of no or Low Read Coverage

Regions of no or low read coverage can be caused by either ineffective sequence capture or inaccuracy in short reads mapping. GC content can affect Solexa base calling and PCR amplification. In addition, low complexity regions can affect short reads mapping. GC content and read coverage were computed in an 81-bp window.

### Prediction of Functional Consequences of SNPs

The final filtered SNPs were annotated using ANNOVAR [45] to identify the gene region to which the SNPs located. The online tool SNPnexus was then used for functional annotation for all the SNPs. SNPnexus is a database providing a complete set of functional annotations of SNPs including consequences to genes and regulatory elements [46]. PolyPhen [47], SIFT [48]–[50] and PROVEAN [51] were used for prediction of functional effect of human nsSNPs. Another online tool, FastSNP [52], was used to predict exonic splicing enhancer/silencer motifs changed by the SNP alleles by integrating results from ESEfinder [53], RESCUE-ESE [54] and FAS-ESS [55] and transcription factor binding sites alteration by TFSEARCH. Moreover, all the SNPs were searched against RegulomeDB to determine involvement in regulatory DNA elements including regions of DNAase hypersensitivity, binding sites of transcription factors, and promoter regions obtained from public ChIP-seq datasets [56].

### SNP Genotyping

SNP Genotyping Using the Sequenom MassARRAY iPLEX Platform was conducted by the Beijing Genomic Institute (BGI, Shenzhen) according to the manufacturer’s protocol, as previously described [57]. Briefly, PCR primers and extension probes were designed using the Sequenom MassARRAY Assay Design 3.0 software. PCR amplification and single-base extension were performed. The purified extension products were dispensed onto a Spectro-CHIP bioarray and analyzed using a MALDI-TOF mass spectrometer. The data were analyzed using the MassARRAY Workstation software (version 3.3). Laboratory personnel conducting the genotyping were blinded to patient information. Strict quality control measures were implemented during genotyping with over 98% concordance between samples genotyped in duplicate.

### Statistical and Data Analysis

Overall survival analysis was conducted using Stata 12. The overall survival time was defined as the month from initial surgery to death from any cause. The relationship between the survival time and an allele was assessed using the Kaplan-Meier method and plotted using Stata 12. All patients alive or lost were censored for analysis. Hazard ratios (HRs) and 95% confidence intervals (CIs) were estimated from a multivariate Cox proportional hazards model, adjusting for age, gender, tumor position, differentiation, tumor stage, positive lymph node status, and infiltration depth, adopting three genetic models (dominant, recessive, and additive). The Hardy-Weinberg equilibrium (HWE) for each SNP was determined using the hwsnp function in Stata 12.

### Confirmation of the Variations

To confirm SNPs identified by the next-generation sequencing techniques, we performed allele-specific PCR (AS-PCR) [18], [19] in a cohort of 46 samples of individual colorectal cancer samples and 4 healthy volunteers. We employed AS-PCR, which is an efficient procedure for genotyping single nucleotide polymorphisms developed by Ye *et al.* [18], [19]. In brief, primers (Table S4) were designed using an online program for designing AS-PCR primers (http://cedar.genetics.soton.ac.uk/public_html/primer1.html) [19]. Each PCR reaction was performed in a total volume of 10 µl, containing a final concentration of 1× TAKARA Ex Taq Buffer, 2.5 mM Mg^2+^, 250 µM of each dNTP,0.2 µM of each primer and 5 U/µl TaKaRa Ex Taq polymerase. The results were analyzed using 2% agarose gel electrophoresis.

## Supporting Information

Figure S1 **The landscape of the two SNPs viewed by the UCSC genome browser.** (A) rs3106189 at the 5′ UTR of TAPBP. (B) rs1052918 at the 3′ UTR of TCF3. The red boxes represent the target SNP sites. Each row represents a regulatory element in UCSC genome browser such as the surrounding SNPs, binding region from ChIP-seq by ENCODE project and conservation scores.
(TIF)

Table S1 **List of SNPs identified in CRC patients.**
(XLSX)

Table S2 **List of SNPs locating in conserved transcription factor binding sites.**
(XLSX)

Table S3 **List of SNPs locating in CpG islands.**
(XLSX)

Table S4 **List of primers used for SNP validation.**
(XLSX)

Table S5 **SNP validation of 8 randomly selected SNPs.**
(XLSX)

Table S6 **SNP Genotyping using the Sequenom MassARRAY iPLEX platform in 30 CRC patients.**
(XLSX)

Table S7 **SNP genotyping using the Sequenom MassARRAY iPLEX platform in 117 CRC patients.**
(XLSX)
