# Supplementary figures and images for: Targeted Re-Sequencing Identified rs3106189 at the 5′ UTR of TAPBP and rs1052918 at the 3′ UTR of TCF3 to Be Associated with the Overall Survival of Colorectal Cancer Patients
Source: PLoS One. 2013 Aug 5;8(8):e70307. doi: 10.1371/journal.pone.0070307 (PMC3734069; doi:10.1371/journal.pone.0070307)

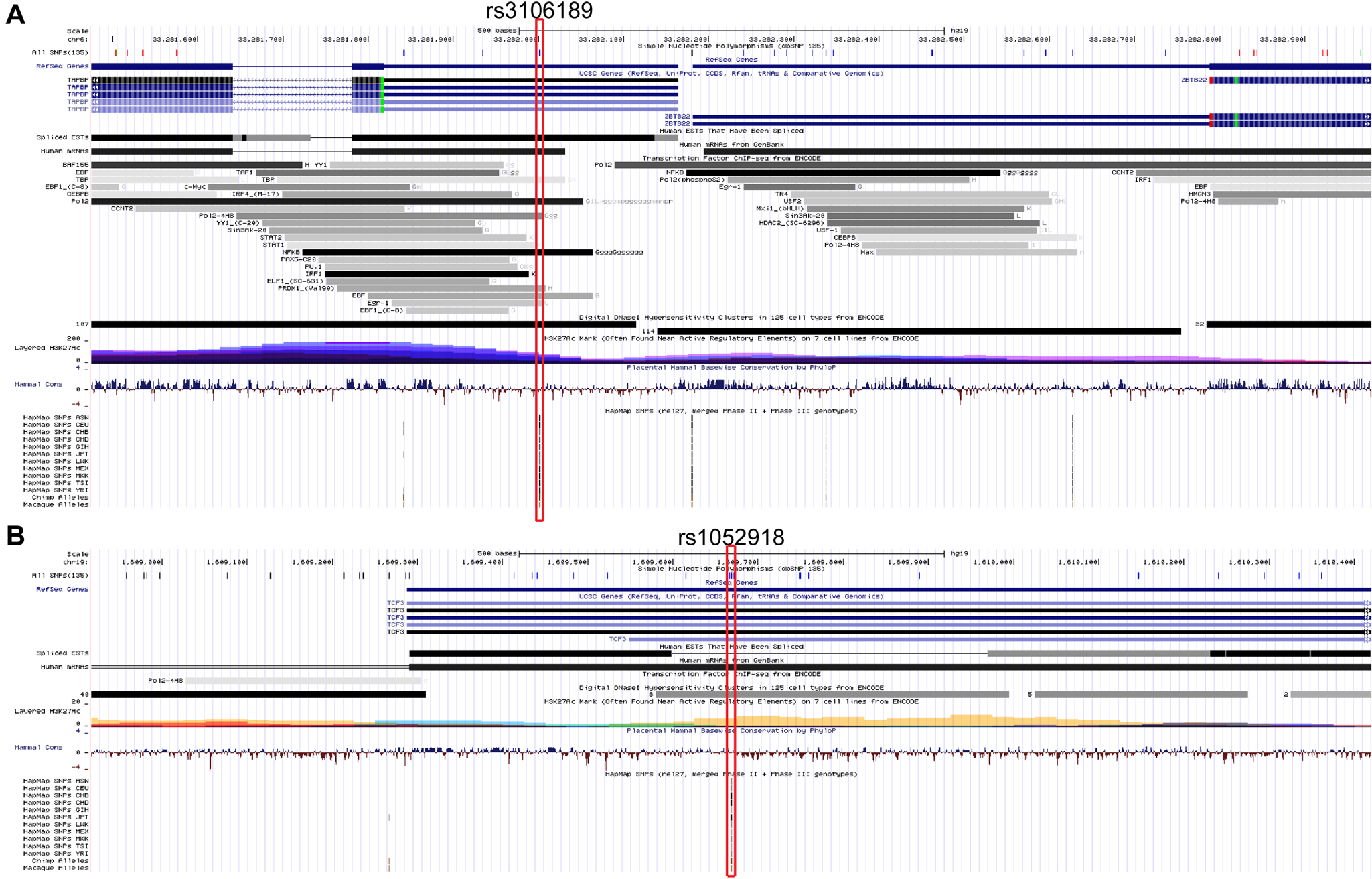

Supplement: Figure S1 — The landscape of the two SNPs viewed by the UCSC genome browser. (A) rs3106189 at the 5′ UTR of TAPBP. (B) rs1052918 at the 3′ UTR of TCF3. The red boxes represent the target SNP sites. Each row represents a regulatory element in UCSC genome browser such as the surrounding SNPs, binding region from ChIP-seq by ENCODE project and conservation scores. (TIF) [file pone.0070307.s001.tif]
